# Prevalence and pattern of alcohol consumption during pregnancy in the Netherlands

**DOI:** 10.1186/s12889-015-2070-1

**Published:** 2015-07-29

**Authors:** Caren I. Lanting, Paula van Dommelen, Karin M. van der Pal-de Bruin, Jack Bennebroek Gravenhorst, Jacobus P. van Wouwe

**Affiliations:** Departments of Child Health and Life Style, Netherlands Organization for Applied Scientific Research TNO, PO Box 3005, 2301 DA Leiden, The Netherlands

**Keywords:** Pregnancy, Alcohol, Smoking, Preconception care

## Abstract

**Objective:**

To estimate the prevalence of alcohol consumption during pregnancy in the Netherlands in 2007 and 2010.

**Method:**

During two identical, nation-wide surveys in 2007 and 2010, questionnaires were handed out to mothers of infants aged ≤6 months who visited a Well-Baby Clinic. By means of the questionnaire mothers were, in addition to questions on infant feeding practices and background variables, asked about their alcohol consumption before, during and after pregnancy. Logistic regression analyses were used to look into relationships of alcohol consumption with maternal and infant characteristics.

**Results:**

We obtained 2,715 questionnaires in 2007, and 1,410 in 2010. Within 6 months before pregnancy, 69 % of women consumed alcohol (data from 2010). During pregnancy 22 % consumed alcohol in 2007, 19 % in 2010. During the first three months of pregnancy, 17 % (2007) and 14 % (2010) of mothers consumed alcohol. Alcohol consumption was mainly one glass (~10 g alcohol) on less than one occasion per month. Compared to 2007, in 2010 more women consumed 1–3 or >3 glasses alcohol per occasion (resp. 11 % to 7 % and 1.4 to 0.7 %). Older women and those with a higher education consumed more alcohol, as did smokers. Birth weight, gestational age and weight for gestational age were not associated with alcohol consumption. In 2007 and 2010, 2.5 % resp. 2.4 % of pregnant women both smoked and consumed alcohol; resp. 70 % and 75 % did neither.

**Conclusion:**

In contrast to Dutch guidelines which advice to completely abstain from alcohol, one in five women in the Netherlands consume alcohol during pregnancy.

## Background

Prevalences of maternal alcohol consumption during pregnancy differ: in a European setting from 5.5 % in Sweden, 13.6 % in Germany and percentages between 12 % and 63 % for France [1–3]. Studies from Dutch cities like Amsterdam (2003) and Rotterdam (2002–2005) estimate these percentages to be resp. 36 % [2] and 37 % [4]; no nation-wide prevalence for the Netherlands is known. Alcohol is the most common teratogen. Consumption during pregnancy is associated with a range of adverse outcomes for the developing infant, most notably Fetal Alcohol Syndrome (FAS) [5–7]. Not all children of mothers who drink alcohol during pregnancy show all symptoms of FAS, but more and more evidence comes up that even low levels may cause developmental problems in later life [8–10]. Therefore, health services in the Netherlands advise pregnant women and women who consider pregnancy to fully abstain from alcohol [11]. This is in line with policies in other countries, like for example Sweden [1].

Adverse effects of alcohol consumption during pregnancy are proportional to the average consumption, the number of glasses consumed on each occasion and the trimester of pregnancy during which it has been consumed [8]. The objective of our study is to determine the prevalence and pattern of alcohol consumption during pregnancy. This is the first population based survey on alcohol consumption during pregnancy in the Netherlands. Furthermore, we investigate associations of alcohol consumption with maternal characteristics and educational level, smoking and birth characteristics.

## Methods

### Data collection

Between 1990 and 2015 several nation-wide cross-sectional studies among mothers of infants aged six month or younger were conducted during Well-Baby clinic visits [12]. Data were collected by means of a two-step procedure. Firstly, for each survey, organizations executing the Mother and Child Health Care program in the Netherlands were asked to provide names and addresses of five Well-Baby Clinics willing to participate. In our invitation to the organizations it was asked to identify Well-Baby Clinics whose clients vary in socioeconomic status. Next, each participating Well-Baby Clinic randomly distributed anonymous questionnaires with stamped, pre-addressed return envelopes to the first 20 visiting mothers of infants under six months of age. In 2007 and 2010, survey questions were included concerning alcohol consumption and smoking habits before, during and after pregnancy. The combined samples of 2007 and 2010 allowed us to study the overall pattern and find changes over time. Mothers were asked to complete the questionnaire at home and to send it back anonymously in a pre-paid envelope. The study design was in agreement with the Helsinki Declaration. The study design was approved by the Leiden University Medical Centre Medical Ethics Committee. Return of the anonymous questionnaire was accepted as implied informed consent, therefore the Committee did not require informed consent otherwise for these cross-sectional surveys.

The questionnaire asked about maternal alcohol consumption within six months before pregnancy (recognition), during pregnancy and the first six months after pregnancy (yes/no); how many occasions (daily/weekly/monthly/hardly) and the amount consumed per occasion (<1/1-3/3-6/>6 glasses alcohol per occasion). These questions in Dutch were pretested and proven to be well understood by responders. As a proxy for SES, formal educational status of mothers was asked and categorized into three levels: low (no or primary education), intermediate (secondary education), or high (higher-professional or university education). Also we asked about smoking status (smoker/non-smoker).

In addition, in the questionnaire, mothers gave their age (in years), civil state (single mother or with partner), the infant’s place of birth (home or hospital delivery), recorded parity (1/2/3/≥4), gestational age (in complete weeks), birth weight (in gram) and gender (boy/girl) of the infant. Gestational age was categorized into extremely preterm (≤28 pregnancy weeks), very preterm (29–31 weeks), moderately preterm (32–36 weeks) and term (≥37 weeks) births. Infants whose birth weight was below the 10th percentile at the corresponding gestational age and gender-specific growth references were classified as Small for Gestational Age (SGA).

### Data analysis

First, we performed unweighted descriptive analyses of mothers who consumed alcohol during and after pregnancy and mothers who did not drink alcohol. Weighted differences between groups were assessed by means of Student’s t, Mann–Whitney or chi-square tests, wherever appropriate. Secondly, associations of alcohol consumption with maternal educational level, smoking during pregnancy and neonatal characteristics were studied by means of (unweighted) logistic regression. Strengths of associations were presented as crude and adjusted odds ratios (OR) and 95 % confidence intervals (95 % CI). During the analyses, adjustments were made for year of the survey (2007 or 2010), maternal age and education, civic state, parity, place of birth (home or hospital), and the child’s gender and smoking during pregnancy. Presented *p*-values were two-sided; *p*-values <0.05 were classified as statistically significant. All analyses were performed with SPSS 22.0.

## Results

### Study population

Questionnaires were handed out by health care workers nation-wide in 222 Well-Baby Clinics. We obtained 2,759 questionnaires of the 5,410 questionnaires handed out in 2007 and 1,448 of the 3,620 in 2010, response rates were respectively 51 % and 40 %. The questions on alcohol use were answered by 2,715 (2007) resp. 1,410 women (2010). Our sample was representative for women who gave birth these years with respect to maternal age at child birth, gestational age, and number of children and place of birth (home or hospital), but not for level of education. In order to obtain representative samples of mothers living in the Netherlands, data were weighted. Weighting factors were set with reference to data of the Dutch population provided by Statistics Netherlands [13]. Mean birth weight ± SD in our sample was 3,477 ± 574 g, mean duration of pregnancy 39.5 weeks, 5 % of infants were born preterm (<<1 % extremely, <1 % very and 4 % moderately preterm), 6 % of infants were born SGA. Mean age of the mothers was 30.6 years (57.9 %; range 15 till 46 years), most are of first parity with a high education level. The questionnaire was filled in when their infants were on average three months old.

Table 1 shows the prevalence rates of alcohol consumption around pregnancy. Sixty-nine percent of women consumed alcohol within 6 month before pregnancy (data from 2010 only). During pregnancy the prevalence of alcohol consumption was 22 % in 2007, and 19 % in 2010 (adj. OR 0.7; 95 % CI 0.6-0.8), (*p* = 0.018). During the first three months of pregnancy, the prevalence was 17 % in 2007, and 14 % 2010 (*p* = 0.026). The first month after birth, percentages were 62 % in 2007, and 48 % in 2010 (*p* < 0.001).

**Table 1:** Prevalence of alcohol consumption before, during and after pregnancy from national surveys of Dutch women conducted in 2007 and 2010

	2007		2010	
	n = 2,759		n = 1,448	
	%	(n)	%	(n)
**Before** *within six months before pregnancy (recognition)*	n.a.		68.9	(998)
**During**	22.4	(618)	19.2*	(278)
*During first three months*	16.5	(455)	13.8**	(200)
*On number of occasions*				
<1 per month	11.1	(306)	9.2	(133)
1-3 per month	4.1	(113)	3.5	(51)
Weekly	1.2	(33)	0,9	(13)
Daily	<0.1	(2)	<0.1	(1)
*Alcohol per occasion*				
Less than one glass	9	(248)	0,4	(6)
One to three glasses	7	(193)	10***	(145)
Three to six glasses	0.7	(19)	1.3***	(19)
More than six glasses	0.4	(11)	0.4	(6)
**After** *the first month after delivery*	62.0	(1,711)	48.2***	(678)

n.a.: not available

*significantly different in 2007 compared to 2010 (*p* = 0.018), **(*p* = 0.026), ***(*p* < 0.001)

The median frequency of alcohol consumption during pregnancy in both surveys (2007 and 2010) was <1 occasion per month (2007: 11 % vs 2010: 9 %). Roughly 4 % drank on 1–3 and only 1 % on ≥4 occasions per month, while three women indicated to consume alcohol daily. Per occasion, most pregnant women consumed <1 glass alcohol or 1–3 glasses, a 0.9 % minority consumed >3 glasses per occasion. The percentage of women who reported binge drinking (>6 glasses alcohol per occasion) during pregnancy was 0.4 % of all pregnant women. In this respect, comparison between 2007 and 2010 showed that the amount of alcohol consumed per occasion had significantly increased (*p* < 0,001). The group of women who consumed 1–3 or >3 glasses of alcohol per occasion had doubled between 2007 and 2010, while binge drinking had not increased.

Alcohol consumption of pregnant women stratified by level of formal education (low, medium, high) and age (<30, ≥30 years) is shown in Table 2. The percentage of women that consumed alcohol during pregnancy increased with the level of education (*p* < 0.001). In the highest educated group 56 % consumed alcohol versus 8 % in the lowest educated group (adj. OR 2.39; 95 % CI 1.80-3.18). A similar association was found for maternal age (*p* = 0.003); the prevalence of alcohol consumption is higher among women aged 30 years or older than among younger women (adj. OR 1.47; 95 % CI 1.25-1.74). Smokers, as compared to non-smokers, had a higher risk also to drink alcohol during pregnancy (adj. OR 2.05; 95 % CI 1.55-2.72).

**Table 2:** Characteristics of women included in 2007 and 2010 (pooled data n = 4,207), stratified by alcohol consumption during pregnancy

	Consuming alcohol (n = 874)	Not consuming alcohol (n = 3,207)	OR (95 % CI) crude	OR (95 % CI) adjusted^a^
Age, mean and *SD* in years	31.25 *4.86*	30.45 *4.68*		
	% (n)	% (n)		
<30 year	35.2 (308)	43.0 (1,381)	1	1
≥30 year	64.8 (566)	58.1 (1,869)	1.53 (1.31-1.78)	1.47 (1.25-1.74)
Maternal education (not weighted)				
Low	7.8 (79)	13.4 (416)	1	1
Medium	36.1 (316)	44.4 (1,376)	1.40 (1.07-1.82)	1.50 (1.10-1.93)
High	56.1 (490)	42.2 (1,312)	2.28 (1.76-2.96)	2.39 (1.80-3.18)
Parity 1th child	46.7 (408)	45.4 (1,459)	1	1
2nd child	36.8 (322)	37.9 (1,219)	0.81 (0.70-0.95)	0.79 (0.67-0.93)
3rd child	10.7 (94)	10.6 (342)	1.09 (0.86-1.37)	1.02 (0.80-1.31)
≥4th child	5.8 (51)	6.1 (195)	0.80 (0.55-1.16)	0.83 (0.60-1.24)
Nonsmoking	88.2 (737)	90.8 (2,867)	1	1
Smoking	11.8 (99)	9.2 (290)	1.43 (1.10-1.85)	2.05 (1.55-2.72)

^a^adjustments were made for the other variables in the table: maternal age and education, parity, smoking and year of the survey

We found no significant association between alcohol consumption during pregnancy as explanatory variable and birth weight, gestational age or weight-for-gestational age. Mothers of second-born infants less often consumed alcohol during pregnancy as compared to mothers who had their first child (adj. OR 0.79; 95 % CI 0.67-0.93).

## Discussion and conclusions

The prevalence of alcohol consumption during pregnancy over 2007 and 2010 in two cross-sectional nation-wide samples of Dutch women is 22 % in 2007 and 19 % in 2010. One in five women do drink alcohol while pregnant. Most of the pregnant women drank alcohol at one occasion per month or less often. Prevalence of alcohol consumption was lower in 2010 compared to 2007 (*p* = 0.018); fewer women consumed alcohol, however the amount consumed per occasion had increased in 2010; one to three glasses of alcohol per occasion compared to less than one glass in 2007 (Table 1). Higher educated and older pregnant women were more likely to consume alcohol before, during and after pregnancy, as were women who smoke. During pregnancy of a 2nd baby, women seemed less likely to consume alcohol (Table 2). In our study, gestational age and birth weight were not associated with alcohol consumption during pregnancy, other features (symptomatic for FAS) have not been studied.

Our data were collected retrospectively and could have been subject to differential misclassification because of recall bias. On the other hand, the inquiry was primarily initiated to explore milk feeding practices and casually addressed alcohol consumption, so that more reliable estimates might have been obtained. Despite of the relatively low response of 40-51 % (although reasonable for this type of study, with no means of sending reminders to potential respondents), our data were representative for the Dutch population with respect to maternal age at child birth, gestational age and number of children and place of birth (home or hospital) and weighted for maternal education. For comparison, the Amsterdam based ABCD-study reported alcohol consumption in 36 % of pregnant women in 2003 [2]. The Rotterdam based generation R study enrolled women between 2002 and 2005 and found 51 % used alcohol in early pregnancy and 37 % continued after pregnancy recognition [4]. In 2007 we found a lower prevalence and even lower in 2010. These lower prevalences may reflect a trend over time of decreased prevalence of alcohol consumption during pregnancy, although differences in study designs and methodologies used may also have had some impact.

In Western Europe, prevalences of alcohol consumption during pregnancy differ between Sweden, 5.5 %, Germany 13.6 %, our study in the Netherlands 19-20 % and in France percentages between 12 % and 63 % [1–3]. Differences in prevalences of alcohol consumption during pregnancy might be related to the overall alcohol consumption in these countries. According to the WHO overall consumption by women aged 15+ years varied less in these countries: in Sweden, Germany, the Netherlands and France resp. 5.5, 7.0, 6.0 and 7.1 L pure alcohol are consumed yearly [14]. Consumption in Germany during pregnancy also increased with higher age and educational level as in our study [2]. And last but not least, advice given to pregnant women vary between countries and may also be responsible for differences between countries.

We have not asked the type of drink in our questionnaire. From other studies we know that the majority of women in the Netherlands drink beer or wine [11]. A glass of wine contains 12 mL of pure alcohol, a glass of beer 12.5 mL (resp. 9.6 and 10 g of alcohol), therefore we assume the amount of alcohol to be 10 g alcohol per glass. The amount of alcohol consumed per occasion and the precise moment in pregnancy both determine the extent of damage to the developing infant. Conception and early pregnancy are most critical moments. Before pregnancy (recognition) the majority of women in our sample consumed alcohol (69 %), in the population of similar age and sex 78 % [13]. Roughly three-quarters of women stopped alcohol consumption on pregnancy recognition, prior to this some drank at levels posing a risk [15]. The recommendation to avoid alcohol during pregnancy may promote anxiety and feelings of guilt in women who have consumed alcohol before recognition. Risk awareness should therefore be taught already at school age or by computer-tailored interventions offered through midwives. A recent trial has been proven effective in stopping and reducing prenatal alcohol use [16].

Alcohol interferes with folate metabolism and reduces its bioavailability. A daily alcohol intake of >3 glasses causes folate deficiency [17], a risk for neural tube defects. In a large population-based study lower prenatal folate status during pregnancy also appears to impair behavioral development; it is associated with emotional problems in 3y old children [18]. In the Netherlands, women of reproductive age are advised to take daily folic acid supplements 4 weeks before conception until 8 weeks after conception. Only half of the women use folic acid supplements in the conception period [18], those higher educated more often. We have no data on folic acid supplementation in our study sample. During the first three month of pregnancy alcohol consumption is less frequent, and in 2010 even lower compared to 2007 (14 % vs 17 %), however the amount of alcohol consumed has increased in 2010 (from <1 glass to 1–3 glasses of alcohol). Binge drinking (>6 glasses per occasion), mostly once per month, is reported in 0.4 % of our sample for both years. These women (a total of 17 in our surveys) appear to be at risk for folate deficiency [17]; identifying these women in clinical practice for targeted intervention may be of benefit.

Another substance with harmful effects on the developing infant is nicotine (cigarettes). In data from earlier surveys we found decreasing prevalences of smoking during pregnancy between 2001 and 2010. The prevalence of smoking differs strongly between women with different levels of education [19], being highest in those lower educated. We found that 2.5 % of the Dutch women both smokes and drinks during pregnancy, around 7 % smokes and does not consume alcohol, around 18 % consumes alcohol (numbers are slightly lower in 2010 compared to 2007). In 2007 70 % and in 2010 75 % of the pregnant women report they neither smoke nor consume alcohol. The combined effects on growth and development of both nicotine and alcohol are severe [6]; one in every 40 Dutch neonates of mothers who both smoked and drank enters life with lower gestational age and birth weight, growth retardation, respiratory disease, central nervous system abnormalities as well as behavioral and cognitive dysfunctions later in life.

In order to decrease this number, it is important to make women of childbearing age aware about risks of alcohol consumption (especially those higher educated) and smoking (especially those lower educated) during pregnancy, as well as benefits of folic acid supplement and breastfeeding. Preconception care can reduce infant mortality and morbidity. Since one in ten of pregnant women smoke and one in five consume alcohol, a considerable number of Dutch neonates’ health and quality of life can improve with an effective preconception care program that educates women of childbearing age about the risks of alcohol consumption and smoking.

## References

[1] Skagerström J, Alehagen S, Haggström-Nordin E, Årestedt K, Nilsen P (2013). Prevalence of alcohol use before and during pregnancy and predictors of drinking during pregnancy: a cross sectional study in Sweden. BMC Public Health.

[2] Pfinder M, Kunst AE, Feldmann R, van Eijsden M, Vrijkotte TG (2013). Preterm birth and small for gestational age in relation to alcohol consumption during pregnancy: stronger associations among vulnerable women? Results from two large Western-European studies. BMC Pregnancy Childbirth.

[3] Dumas A, Simmat-Durand L, Lejeune C. Pregnancy and substance use in France: A literature review. J Gynecol Obstet Biol Reprod (Paris) 2014. doi: 10.1016/j.jgyn.2014.05.008 10.1016/j.jgyn.2014.05.008 24930726

[4] Bakker R, Pluimgraaff LE, Steegers EAP, Raat H, Tiemeier H, Hofman A, Jaddoe VWV (2010). Associations of light and moderate maternal alcohol consumption with fetal growth characteristics: The Generation R Study. Int J Epidem.

[5] Ungerer M, Knezovich J, Ramsay M (2013). In utero alcohol exposure, epigenetic changes and their consequences. Alcohol Res.

[6] Behnke M, Smith V (2013). Prenatal substance abuse; short- and long-term effects on the exposed fetus. Pediatrics.

[7] Mukherjee RAS, Turk J (2004). Fetal Alcohol Syndrome. Lancet.

[8] Sood B, Delaney-Black V, Covington C, Nordstrom-Klee B, Ager J, Templin T (2001). Prenatal exposure and childhood behavior at age 6 to 7 years: 1. Dose–response effect. Pediatrics.

[9] Day NL, Helsel A, Sonon K, Goldschmidt L (2013). The association between prenatal alcohol exposure and behavior at 22 years of age. Alcohol Clin Exp Res.

[10] Flak AL, Su S, Bertrand J, Denny CH, Kesmodel US, Cogswell ME (2014). The association of mild, moderate, and binge prenatal alcohol exposure and child neuropsychological outcomes: a meta-analysis. Alcohol Clin Exp Res.

[11] Health Counsel of the Netherlands. Risk of alcohol consumption related to conception, pregnancy and breastfeeding. 2005;2004/22.

[12] Lanting CI, Van Wouwe JP, Reijneveld SA (2005). Infant milk feeding practices in the Netherlands and associated factors. Acta Paediatr.

[13] Statistics Netherlands: statline.cbs.nl/Statweb/publication/?DM = SLEN&PA = 81177eng&D1 = 9-13&D2 = 0,2-12,26-38&D3 = 0&D4 = 0&LA = EN&HDR = T,G3&STB = G1,G2&VW = T, consulted May 21st 2015.

[14] WHO Global status report on alcohol and health 2014 www.who.int/substance_abuse/publications/global_alcohol_report/en/, consulted June 2nd 2015.

[15] Parackal SM, Parackal MK, Harraway JA (2013). Prevalence and correlates of drinking in early pregnancy among women who stopped drinking on pregnancy recognition. Matern Child Health J.

[16] Van der Wulp NY, Hoving C, Eijmael K, Candel MJJM, Van Dalen W, De Vries H (2014). Reducing alcohol use during pregnancy via health counseling by midwives and internet-based computer-tailored feedback: a cluster randomized trial. J Med Internet Res.

[17] Medici V, Halsted CH (2013). Folate, alcohol and liver disease. Mol Nutr Food Res.

[18] Steenweg-de Graaff J, Roza SJ, Steegers EAP, Hofman A, Verhulst FC, Jaddoe VWV, Tiemeier H (2012). Maternal folate status in early preganacy and child emotional and behavioral problems: the Generation R Study. Am J Clin Nutr.

[19] Lanting CI, Buitendijk SE, Crone MR, Segaar D, Bennebroek Gravenhorst J, van Wouwe JP (2009). Clustering of socioeconomic, behavioural, and neonatal risk factors for infant health in pregnant smokers. PLoS ONE.

